# Deciphering the role of APP in synaptic function

**DOI:** 10.1186/1750-1326-7-S1-L7

**Published:** 2012-02-07

**Authors:** Hui Zheng

**Affiliations:** 1 Huffington Center on Aging, Department of Molecular and Human Genetics, Baylor College of Medicine, Houston, Texas, USA

## Background

Genetic and biochemical studies establish a central role of the amyloid precursor protein (APP) in Alzheimer’s disease (AD): genetic mutations and gene amplification of *APP* are linked to a subset of early onset familial Alzheimer’s disease (FAD), and APP processing generates β-amyloid (Aβ) peptides, which are the principal components of the amyloid plaque pathology. Although β-amyloid plaques are the hallmark of AD, synaptic dysfunction closely correlates with cognitive impairment and is recognized as a causal event leading to AD pathogenesis. Since Aβ is naturally generated along with other products through APP processing, investigating the role of APP and its cleavage products in synaptic function and dysfunction is critically important in understanding AD pathogenesis.

## Results

We seek to understand the physiological functions of APP in neurons and synapses using *in vivo* mouse models and *in vitro* culture systems. Analysis of the *APP* knockout mice allows us to identify a functional role of APP in synaptic plasticity and learning and memory. Our investigation of mice deficient in *APP* and its homolog *APLP2* establishes an essential role of APP family protein in mediating cholinergic synaptic structure and neurotransmission in both peripheral neuromuscular synapse and central cholinergic neurons. By creating an *APP* conditional allele, we demonstrate that APP is required in both pre- and postsynaptic terminals; and that pre- and postsynaptic APP interact to mediate synaptic structure and function. These *in vivo* findings are supported by *in vitro* mixed-culture studies, which reveal that APP potently induces synaptogenesis. Independent of the synaptic adhesion property that requires the full-length protein, we found that the soluble secreted APP ectodomain mediates transcription of genes related to aging and amyloid sequestration.

## Conclusion

Our studies identify APP as synaptic adhesion and signaling molecules, which are mediated by distinct functional domains. Perturbation of these activities may contribute to synaptic dysfunction and AD pathogenesis.

